# Regulators Associated with Clinical Outcomes Revealed by DNA Methylation Data in Breast Cancer

**DOI:** 10.1371/journal.pcbi.1004269

**Published:** 2015-05-21

**Authors:** Matthew H. Ung, Frederick S. Varn, Shaoke Lou, Chao Cheng

**Affiliations:** 1 Department of Genetics, Geisel School of Medicine at Dartmouth, Hanover, New Hampshire, United States of America; 2 Institute for Quantitative Biomedical Sciences, Geisel School of Medicine at Dartmouth, Lebanon, New Hampshire, United States of America; 3 Norris Cotton Cancer Center, Geisel School of Medicine at Dartmouth, Lebanon, New Hampshire, United States of America; University of Toronto, CANADA

## Abstract

The regulatory architecture of breast cancer is extraordinarily complex and gene misregulation can occur at many levels, with transcriptional malfunction being a major cause. This dysfunctional process typically involves additional regulatory modulators including DNA methylation. Thus, the interplay between transcription factor (TF) binding and DNA methylation are two components of a cancer regulatory interactome presumed to display correlated signals. As proof of concept, we performed a systematic motif-based *in silico* analysis to infer all potential TFs that are involved in breast cancer prognosis through an association with DNA methylation changes. Using breast cancer DNA methylation and clinical data derived from The Cancer Genome Atlas (TCGA), we carried out a systematic inference of TFs whose misregulation underlie different clinical subtypes of breast cancer. Our analysis identified TFs known to be associated with clinical outcomes of p53 and ER (estrogen receptor) subtypes of breast cancer, while also predicting new TFs that may also be involved. Furthermore, our results suggest that misregulation in breast cancer can be caused by the binding of alternative factors to the binding sites of TFs whose activity has been ablated. Overall, this study provides a comprehensive analysis that links DNA methylation to TF binding to patient prognosis.

## Author Summary

DNA methylation is a ubiquitous and simple covalent modification that occurs directly on genetic material whereby a simple methyl group (CH3) is attached to Cytosine nucleotides in the context of CpG sites. Modifications of these sites have been postulated to function in gene regulation, potentially via interactions with transcription factors. In this study, we hypothesized that DNA methylation signals contain valuable information that can help infer transcription factors that may be associated with a given disease. Here, we utilize the vast repository of breast cancer data that is available in the public domain, and which contains a rich resource for DNA methylation and clinical data on breast cancer patients. In this guilt-by-association analysis, we postulated that conserved transcription factor binding motifs that are statistically enriched in regions near methylated CpG sites that are correlated with breast cancer patient survival would suggest that their cognate transcription factors would play a role in the initiation, growth, metastasis, or even suppression of the tumor. This integrative approach supports the claim that DNA methylation profiling of patient tumors in the clinic may contain valuable information that can guide the development of treatment regimens for individual patients; thus contributing to the progression of precision medicine.

## Introduction

DNA methylation is a critical regulatory process that involves direct chemical modification of genetic material via the addition of a methyl moiety to the 5th carbon of Cytosine nucleotides. These covalent modifications occur most prevalently on CpG dinucleotides (CpGs) and are reversible, thus allowing the DNA methylome to achieve a balance of stability and plasticity. DNA methylation plays essential roles in X-chromosome inactivation [1], genomic imprinting [2], transposable elements silencing [3], stem cell differentiation [1,4–6], embryonic development [7,8], and inflammation [9,10]. Considering these critical roles, aberrant DNA methylation patterning has been observed in nearly all cancer types and in a plethora of non-cancer diseases including autoimmune disorders [11,12], neurological diseases [11,13] metabolic disorders [14], and cardiovascular disease [15]. Furthermore, DNA methylation signatures and markers have been used to stratify cancer subtypes and predict patient prognosis [16–18].

Recently, the use of DNA methylation profiling to predict prognostic outcomes of diseased patients has gained popularity. In breast cancer, studies have shown that ER+ and ER- breast cancer cell lines could be distinguished by examining their DNA methylation patterns. Sun et al. identified 84 genes that were differentially methylated between ER+ and ER- cell lines [19]. Additionally, the TCGA consortium clustered 802 primary breast cancer samples based on their DNA methylation signals; this yielded 5 distinct clusters that comprised samples that exhibited varying molecular phenotypes [20]. In a recent study, Anjum et al. identified a BRCA1 mutation-associated DNA methylation signature in 144 case-control primary blood samples that was predictive of breast cancer incidence and patient prognosis [21]. Furthermore, Bullinger et al. applied a MALDI-TOF-MS based methylation analysis to identify a DNA methylation signature in 182 acute myeloid leukemia primary samples that was predictive of patient outcomes [22]. Several other studies have identified DNA methylation signatures and markers in primary breast tumor samples that were shown to predict patient outcome [23–26]. These studies have shown that understanding DNA methylation patterning and dissecting its functions provide valuable insight into its regulatory roles, which may ultimately introduce new avenues for developing efficacious breast cancer treatments.

Despite recent focus on epigenetic based markers, the exact mechanism(s) by which DNA methylation regulates gene expression has yet to be elucidated but its interaction with transcription factors (TFs) have been shown to be a critical mechanism [27–31]. It has been suggested that 5-methyl-CpGs (5meCpGs) physically impede the binding of TFs to their cognate sequences causing gene silencing [27]. Additionally, 5-meCpGs can indirectly control gene expression by modulating local chromatin structure via recruitment of histone remodeling factors such as histone deacetylases and histone methyltransferases [32–34]. Physical obstruction of TF binding and compaction of chromatin structure suggest that DNA methylation exerts a silencing effect; however, studies have shown TFs such as SP1 can bind 5-meCpGs and induce gene expression [35]. In a comprehensive and systematic genomics study, Hu et al. applied a protein microarray-based approach to identify which of 1321 TFs and 210 co-factors have the capacity to bind motifs containing methylated CpGs [36]. They reported 41 TFs and 6 TF co-factors that bound 5meCpG in a sequence-specific manner [36]. Indeed, it is possible that DNA methylation patterns are just passive markers of TF binding or gene regulation whereby CpGs in unbound chromatin are methylated and have no functional relevance [37,38]. Regardless of the exact mechanism, we postulate that changes in CpG signals near putative transcription factor binding sites (TFBSs) can reflect the activity of TFs and can be used to infer the underlying transcriptional machineries that drive the progression of several subgroups of breast cancer.

We have previously integrated ENCODE [39] and TCGA [20] data to computationally examine the association between TF binding and DNA methylation levels in TFBSs (i.e. ERα) [31]. We found that there is a strong negative correlation between ERα activity and DNA methylation levels within ERα binding sites in breast cancer [31]. More importantly, differentially methylated CpGs between ER+ and ER- breast cancer are enriched in the DNA regions surrounding ERα binding peaks (determined by ChIP-seq) in a distance dependent manner—the closer to the center of binding peaks, the more differentially methylated the CpGs tend to be [31]. Conversely, given a set of differentially methylated CpG sites between ER+ and ER- samples, we would expect the binding site motif of ERα or other functionally related TFs to be enriched within the vicinity of these CpG sites. These findings suggest that DNA methylation patterns and their signals are informative for exploring transcriptional regulation mediated by TFs [14].

In this study, we aimed to utilize DNA methylation data derived from primary breast cancer samples to identify TFs that are associated with patient survival via their relationship with methylated CpGs. To achieve this we connected the DNA methylation-TF interactome to breast cancer patient survival using datasets derived from ENCODE and TCGA [20]. Specifically, we identified a list of CpG sites that were significantly correlated with patient survival time in their methylation level. We then determined which TF binding sites are enriched in DNA regions surrounding survival-associated CpGs to extrapolate TFs that are associated with patient survival. Interestingly, we ascertained that ERα TF binding motifs were significantly enriched in survival-associated CpG regions in ER- samples only, and p53 TF binding motifs were enriched in survival-associated CpGs regions in p53- samples only. Overall, our analysis framework demonstrates the intimate linkage between DNA methylation, TF binding, and breast cancer patient prognosis.

## Results

### Overview of our method for identifying survival-associated TFs

**Fig 1:**
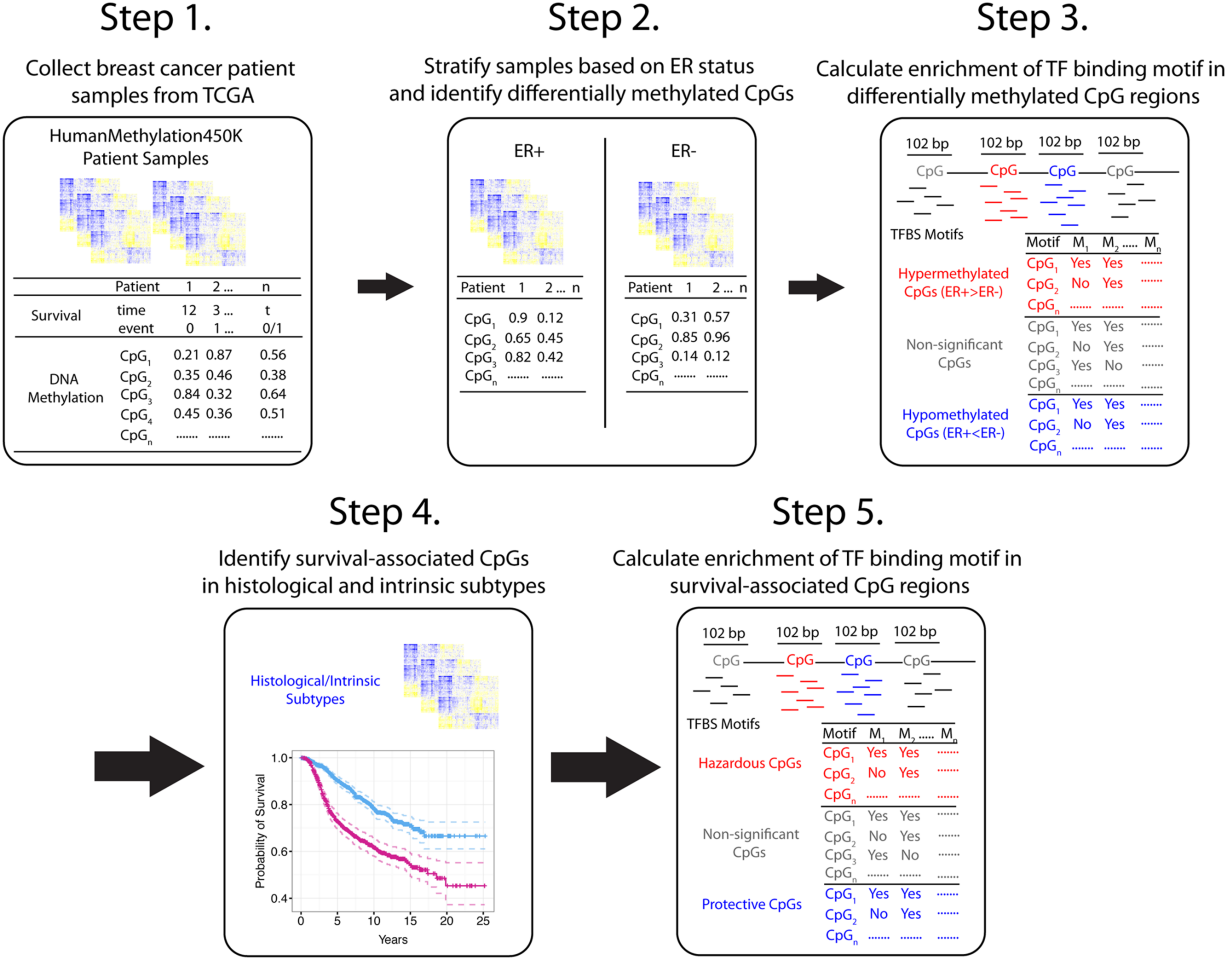
Overview of analysis. To show that TF activity can be inferred from DNA methylation levels, patient samples were stratified on ER status to determine differentially methylated CpGs. Enrichment analysis was then carried out to determine presence of TF binding motifs in differentially methylated CpG regions (Steps 1–3). To infer TFs associated with clinical outcomes, β-values of 376,667 CpGs across (Step 4) 562 samples were individually used as covariates into a univariate Cox proportional hazards model [40]. Statistically significant CpGs were labeled as protective, hazardous, or survival-associated (Pro+Haz). A 102 bp region centered at each significant CpG was then interrogated (after filtering out overlapping CpG regions) for the presence of TF binding motifs (Step 5). TF binding motifs enriched across CpGs were then inferred to be survival-associated via an epigenetic relationship.

The ultimate goal of our analysis was to identify TFs that impact breast cancer patient prognosis via an association with CpG methylation. By identifying TF binding motifs enriched in regions containing differentially methylated CpGs or survival-associated CpGs, we were able to demonstrate a relationship between TF-DNA methylation mediated regulation and overall patient survival rates. Fig 1 depicts our integrated approach to dissecting epigenetic involvement in transcriptional regulation underlying breast cancer patient survival. First, we investigated TF binding motifs enriched in differentially methylated CpG regions to demonstrate that methylation data are informative for inferring transcriptional regulation in breast cancer (Fig 1: Top). We successfully detected the enrichment of ERα TF binding motifs in DNA regions surrounding CpG sites that were differentially methylated between ER+ and ER- breast cancer patients.

Most importantly, we applied motif enrichment analysis to survival-associated CpGs (Fig 1: Bottom) using all breast cancer samples and subsets of samples stratified based on histological, intrinsic, and CpG beta-value intensity phenotypes. We first identified a set of survival-associated CpGs by correlating the methylation levels of each CpG across TCGA breast cancer patient samples using a univariate Cox proportional hazards model [40]. Second, we defined a 102 bp genomic region centered at each CpG (henceforth referred to as a CpG region) and computationally searched for the presence of TF binding motifs in this region. Third, we systematically calculated TF binding motif enrichment in these CpG regions in different subtypes of breast cancer.

### Enriched TF binding motifs in differentially methylated CpG sites between ER+ and ER- breast samples

To preliminarily demonstrate that TF-mediated transcriptional regulation could be inferred from DNA methylation signals, we investigated the relationship between CpGs with altered methylation levels and the presence of putative TF binding motifs vicinal to these CpGs. Specifically, we identified CpGs that were differentially methylated between estrogen receptor positive (ER+) and estrogen receptor negative (ER-) breast cancer samples in TCGA data using a Student’s t-test, and then examined the occurrences of putative TF binding motifs within DNA regions surrounding these CpGs.

**Fig 2:**
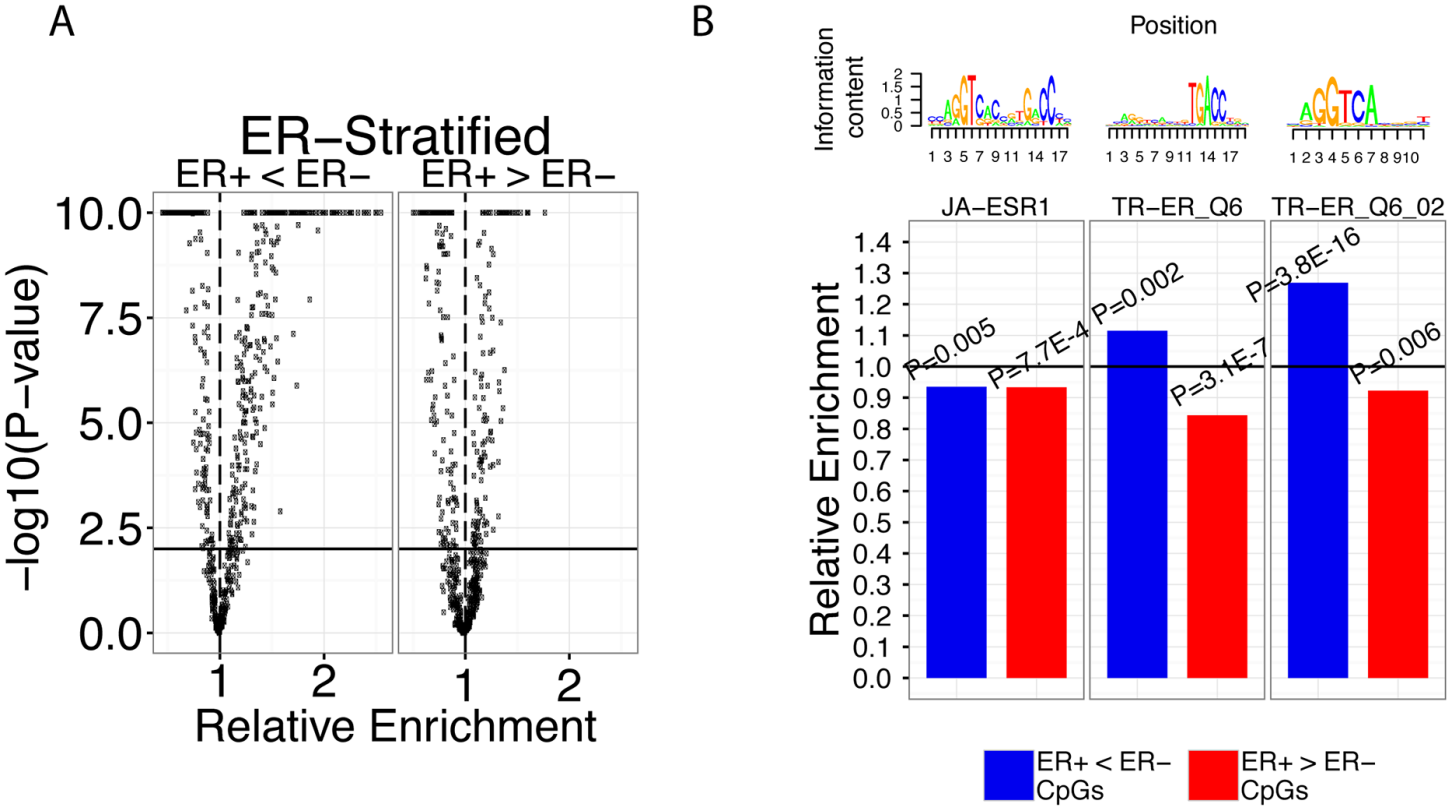
ERα TF binding motifs enriched in differentially methylated CpG regions. **A)** Global comparison of the total number of TF binding motifs enriched in hypo- or hypermethylated and their relative enrichment levels. Horizontal bold line corresponds to P = 0.01 and vertical dashed line corresponds to an enrichment level of 1. -log_10_(p-values) greater than 10 or less than -10 were trimmed at 10 and -10, respectively. **B)** Relative enrichment values of JA-ESR1, TR-ER_Q6, and TR-ER_Q6_02 motifs in hypomethylated (ER+<ER-), hypermethylated (ER+>ER-) CpGs in ER-stratified samples. P-values show significance of enrichment or depletion of the motif in the two categories. Horizontal bold line corresponds to an enrichment level of 1.

We systematically calculated the enrichment levels of 703 TF binding motifs available from the TRANSFAC and JASPAR databases in differentially methylated CpG regions. Our analysis identified 60 TF binding motifs (38 TFs) enriched in and 105 TF binding motifs (67 TFs) depleted in hypomethylated (ER+ < ER-) CpG regions at a P<1E-15 significance threshold (Fig 2A) (adjusted p-value using the Benjamini-Hochberg multiple testing correction method; hereafter, all reported p-values have been adjusted unless otherwise indicated). In addition, we identified 12 TF binding motifs (10 TFs) enriched in and 50 TF binding motifs (35 TFs) depleted in hypermethylated (ER+ > ER-) CpG regions at the same threshold (Fig 2A). Similar results were obtained when a Wilcoxon ranked sum test was used to identify differentially expressed CpGs (S1 Table).

To validate the accuracy of our systematic analysis, we directed our attention to ERα TF binding motifs. Since stratifying breast cancer patient samples into ER+ and ER- groups is analogous to controlling for ERα activity, we hypothesized that ERα TF binding motifs would be significantly enriched in hypomethylated (ER+ < ER-) CpG regions or, alternatively, depleted in hypermethylated (ER+ > ER-) CpG regions. Our data set includes three ERα TF binding motifs: JA-ESR1, TR-ER_Q6, and TR-ER_Q6_02 for which we calculated their enrichment in the two CpG sets (hypo- and hypermethylated sets). First, JA-ESR1 was depleted in hypermethylated CpG regions 0.93-fold (P = 7.7E-4) and depleted in hypomethylated CpG regions 0.94-fold (P = 0.005) (Fig 2). Second, we observed that the TR-ER_Q6 motif was also depleted in hypermethylated CpG regions 0.84-fold (P = 3.1E-7), but unlike JA-ESR1, was enriched 1.11-fold in hypomethylated CpG regions (P = 0.003) (Fig 2B). Lastly, the TR-ER_Q6_02 motif was depleted in hypermethylated CpG regions 0.92-fold (P = 0.006) and enriched in hypomethylated CpG regions 1.27-fold (P = 3.8E-16). These results indicate that TF binding activity can be inferred based on enrichment of their motifs in DNA regions near informative CpGs (e.g. differentially methylated CpGs). Previously, we had established that DNA methylation within ERα binding sites was anti-correlated with *ESR1* expression by integrating TCGA (gene expression and DNA methylation) and ENCODE ChIP-seq data [14]. Therefore, the depletion of all 3 ERα TF binding motifs in hypermethylated CpG regions is in accordance with our previous analysis confirming that ERα activity is associated with loss of binding site-specific DNA methylation [41].

In addition to ERα TF binding motifs, we also identified a number of other TF binding motifs that are known to be associated with ERα. Strikingly, hypomethylated CpG regions contained 18 TF binding motifs (corresponding to 5 FOX family transcription factors, GATA1, HFH8, XFD2, and XFD3) that exhibited greater than 2-fold enrichment, whereas hypermethylated CpG regions contained none (Fig 2A). Operating under the passive model [42], this suggests that loss of methylation is generally associated with enhanced binding activity of these transcription factors in ER+ breast cancer. In addition, we identified all GATA3 and FOXO1 TF binding motifs to be enriched in hypomethylated CpG regions and depleted in hypermethylated regions suggesting that these TFs are associated with ERα activity. Indeed, it has been experimentally shown that FOXA1 influences ERα function by modulating ER-chromatin interactions and *FOXA1* deficiency results in loss of ERα activity (S1 Table) [43–45]. In addition, GATA3 has been shown to be necessary for estradiol stimulation of breast cancer cells and more recently, modulate ERα access to enhancer regions [46,47]. Overall, our motif enrichment results in differentially methylated CpG regions results are consistent with the known biological roles of our identified TFs.

We were additionally able to verify several enriched TF binding motifs via a *de novo* motif search in hyper- and hypomethylated CpG regions. Specifically, we applied the Discriminative Regular Expression Motif Elicitation (DREME) algorithm to identify enriched DNA motifs in hyper- and hypomethylated CpG regions and then matched them to known motifs (See Materials and Methods). We were able to detect the presence of the ESR1 motif in hypomethylated CpG regions but not in the hypermethylated CpG regions, which is consistent with the enrichment results for ERα. We were also able to confirm motifs including FOXO1, SP1, KLF4, EGR2, and E2F1 in hypomethylated CpG regions and TCF3, NHLH1, and HEB in hypermethylated CpG regions.

### Survival-associated TFs in breast cancer

After identifying enriched TF binding motifs in differentially methylated CpG regions, our next objective was to determine if TFs associated with patient survival could be inferred based on DNA methylation signals. Aberrant TF activity and DNA methylation changes have both been known to play a role in carcinogenesis and cancer progression. However, to our knowledge, there has been no other study that has systematically investigated survival-associated TF-DNA methylation relationships at the level of specific TF-CpG interaction. Thus, to proceed with this high-resolution analysis, we pinpointed CpGs whose methylation levels were significantly associated with breast cancer patient survival and calculated the enrichment of TF binding motifs in the regions surrounding these CpGs. CpGs with hazard ratios <1 were categorized as protective, CpGs with hazard ratio >1 were categorized as hazardous, and pooled protective and hazardous CpGs were simply categorized as survival-associated. We hypothesized that survival-associated fluctuations in CpG methylation intensities would be informative to the activity of specific survival-associated TFs.

**Fig 3:**
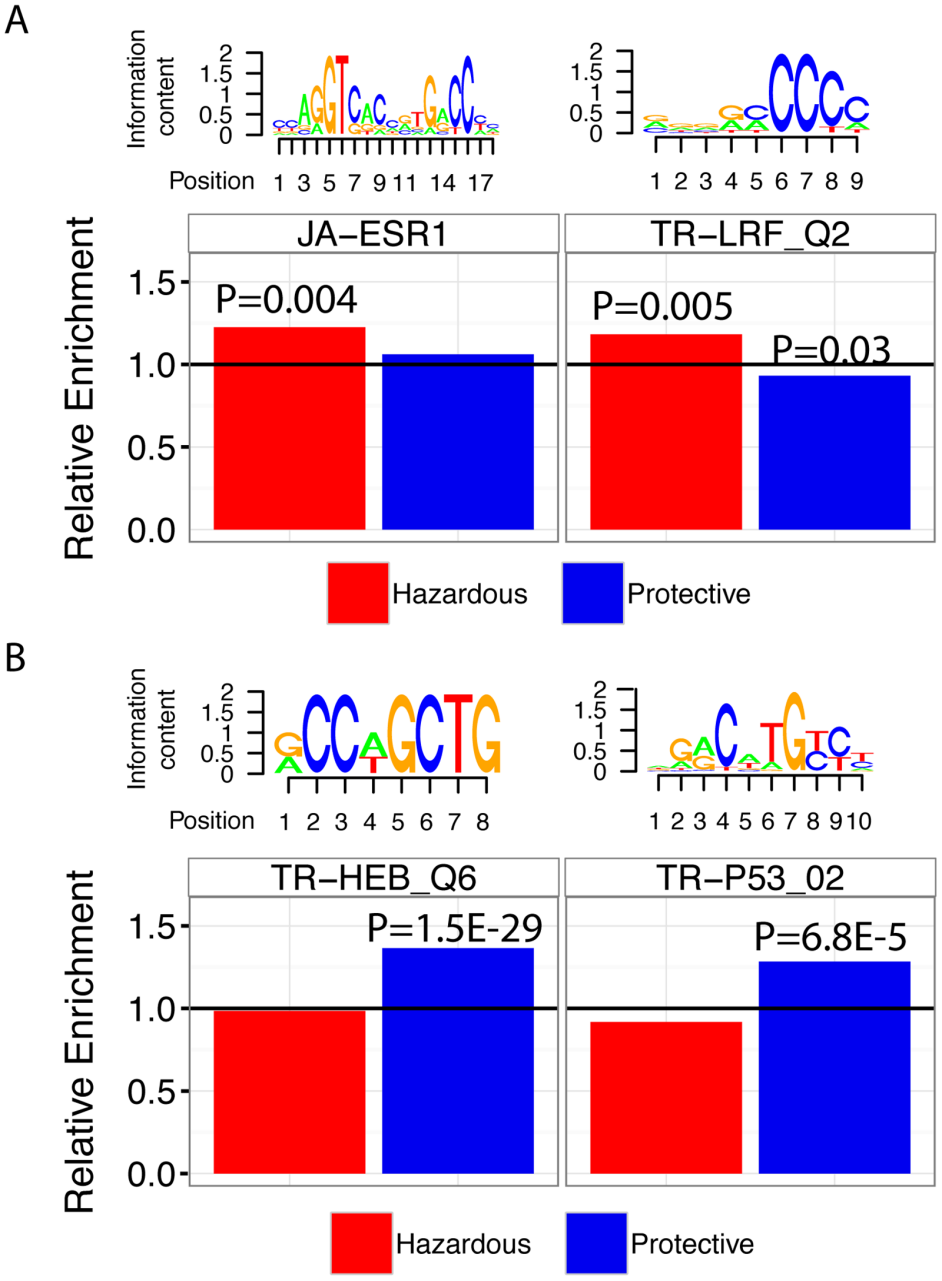
TF binding motifs enriched in survival-associated CpG regions in primary breast cancer samples. **A)** Relative enrichment levels of JA-ESR1 and TR-LRF_Q2 in all breast cancer samples. **B)** Relative enrichment levels of TR-HEB_Q6 and TR-P53_02 in all breast cancer samples. Motif logos are provided above each panel to show information content of each motif.

When survival analysis was implemented using all samples, we were able to identify 92 TF binding motifs (62 TFs) enriched and 143 TF binding motifs (98 TFs) depleted in protective CpG regions at significance level P<0.01 (S2 Table). For hazardous CpG regions, we detected 11 TF binding motifs (9 TFs) enriched and 2 TF binding motifs (2 TFs) depleted at the same threshold, respectively. Fig 3 highlights four examples of TF binding motifs enriched in survival-associated CpG regions: p53, ERα, HEB, and LRF. First, JA-ESR1 exhibited an enrichment score of 1.23 at P = 0.004 in hazardous CpG regions (Fig 3A). This indicates that the effect ERα binding activity—it is known that ER status is a significant clinical factor for predicting survival of breast cancer patients—has on patient survival can be inferred from DNA methylation signals correlated with patient prognosis. Second, LRF is an oncogenic transcription factor involved in cell growth and differentiation, and is known to be overexpressed in breast cancer [48]. Our analysis shows that the TR-LRF_Q2 TF binding motif is enriched 1.18 times in hazardous CpG regions (P = 0.005); additionally, it is depleted 0.93-fold in protective CpG regions (P = 0.03) (Fig 3A). In one study, Maeda et al. reported that LRF is necessary for embryonic fibroblast cells (MEFs) to undergo transformation even when other potent oncogenes such as H-Ras, T-antigen, and MYC are expressed [49]. Third, we identified HEB (TCF12) to be enriched 1.36-fold in protective CpG regions (P = 1.5E-29, Fig 3B). HEB has been previously reported to correlate with colorectal cancer metastasis by inhibiting E-cadherin, thus verifying HEB as a potential oncofactor [50]. Fourth, it is well established that loss of p53 activity is detected in approximately 50% of all cancers [51]. In our analysis we were able to identify all p53 TF binding motifs to be enriched in survival-associated CpGs (P<0.05). To highlight, the TR-P53_02 motif was enriched 1.28-fold in protective CpG regions (P = 6.8E-5) (Fig 3A). This again indicates that DNA methylation levels provide information about the activity of key transcriptional regulators.

To provide a brief summary, we show the top ten significant TF binding motifs enriched or depleted in protective CpG regions in Table 1. Similarly, we illustrate the top ten TF binding motifs enriched or depleted in hazardous CpG regions in Table 2.

**Table 1:** Top 10 TF binding motifs enriched/depleted in protective CpG regions.

TF Binding Motif (PWM)	Transcription Factor	Adjusted P-value (BH)	Enrichment/ Depletion
TR-LBP1_Q6	UBP1	2.34E-36	1.45
TR-ETF_Q6	ETF	2.17E-32	0.75
TR-E2F1_Q3_01	E2F1	2.31E-32	0.68
TR-AP4_Q6_01	TFAP4	3.50E-32	1.40
TR-HEB_Q6	TCF12	1.53E-29	1.36
JA-Myf	MYF5	2.09E-26	1.32
TR-AP4_Q6	TFAP4	7.66E-24	1.35
TR-AP4_Q5	TFAP4	6.57E-22	1.35
TR-MYOD_Q6	MYOD	1.74E-21	1.33
TR-ZF5_01	FP161	1.77E-21	0.73

**Table 2:** Top 10 TF binding motifs enriched/depleted in hazardous CpG regions.

TF Binding Motif (PWM)	Transcription Factor	Adjusted P-value (BH)	Enrichment/ Depletion
JA-EMBP1	EMBP1	0.0012	1.52
JA-MAX	MAX	0.0028	1.40
TR-USF_02	USF1	0.0028	1.60
TR-CDX2_Q5	CDX2	0.0035	0.36
TR-EBOX_Q6_01	EBOX	0.0039	1.25
JA-ESR1	ESR1	0.0045	1.23
TR-SREBP1_01	SREBP1_01	0.0045	1.44
TR-FOXO4_02	FOXO4	0.0045	0.58
TR-MYCMAX_03	MYCMAX	0.0045	1.45
TR-LRF_Q2	FBI1	0.0045	1.18

### TF binding motifs enriched in survival-associated CpGs in ER and p53 stratified breast cancer

**Fig 4:**
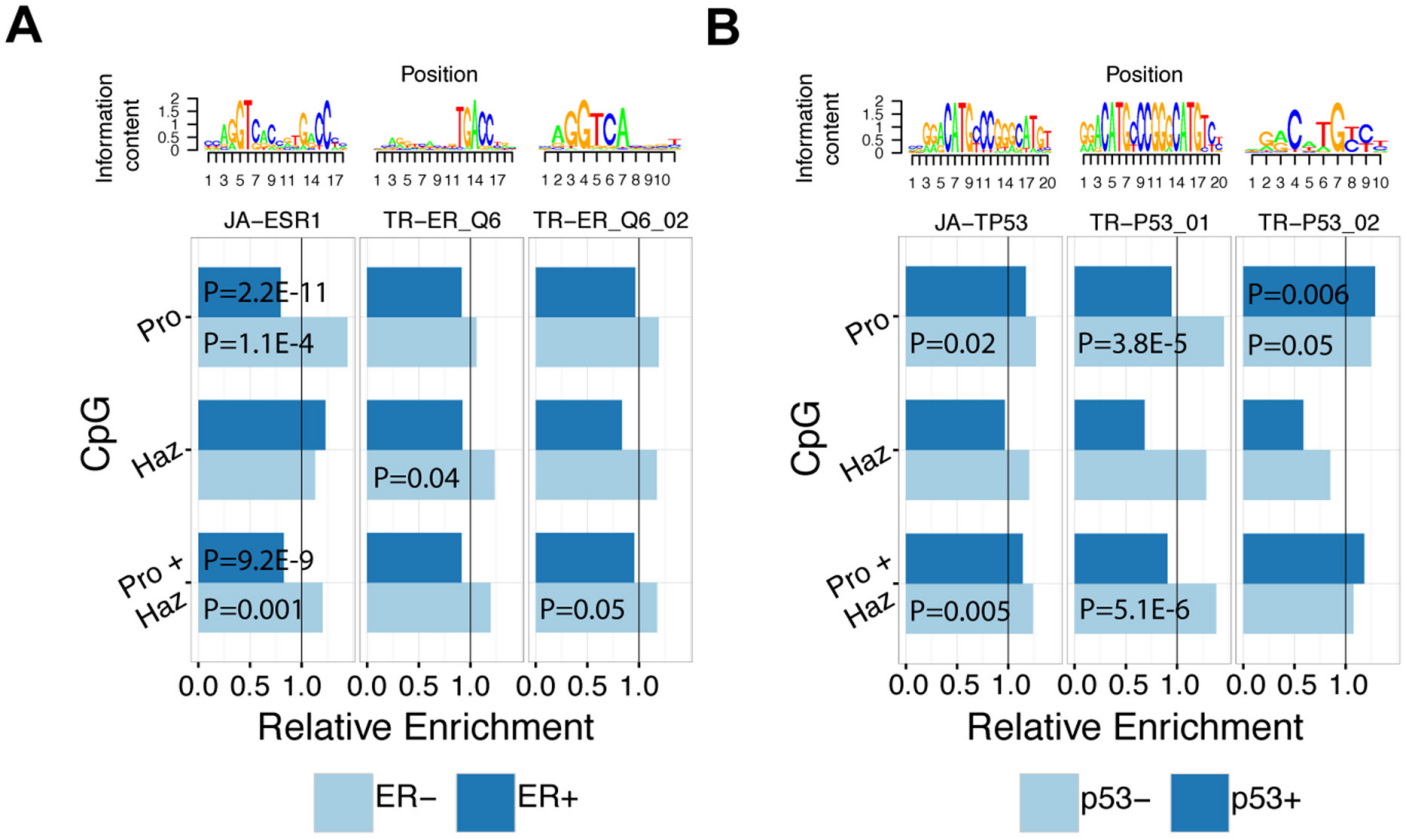
TF binding motifs enriched in survival-associated CpG regions in ER-stratified samples. **A)** Enrichment values of ERα TF binding motifs (JA-ESR1, TR-ER_Q6, TR-ER_Q6_02) in hazardous, protective, and Survival-associated (Pro+Haz) CpG regions in ER+ and ER- samples. **B)** Enrichment values of p53 TF binding motifs (JA-TP53, TR-P53_01, TR-P53_02) in hazardous, protective, and survival-associated (Pro+Haz) CpG regions in p53+ and p53- samples. Vertical line corresponds to an enrichment level of 1.

To demonstrate that survival-associated CpGs are informative for identifying clinically relevant TFs, we focus on two key breast cancer-related proteins: ERα and p53. ERα and p53 are major proteins whose expression levels are typically measured in breast cancer cases to determine the molecular status of the tumor, and it is well-established practice to use this information for determining prognosis and treatment strategies. Here, we explored two major subtyping schemata by systematically calculating the enrichment/depletion of all TF binding motifs (in particular ERα and p53) in survival-associated CpG regions for ER+, ER-, p53+, and p53- breast cancer subtypes (S3–S6 Tables). First, we directed our focus to ERα TF binding in ER-stratified samples and were able to identify JA-ESR1 to be enriched 1.44-fold in protective CpG regions in the ER- subtype but not in the ER+ samples (P = 2.6E-5, Fig 4A). In fact, in ER+ samples, JA-ESR1 is significantly depleted 0.80-fold in protective CpG regions (P = 2.2E-11) (Fig 4A). When protective and hazardous CpGs are combined, JA-ESR1 is enriched 1.20-fold in survival-associated CpGs in ER- (P = 0.001) samples only and depleted 0.83-fold (P = 9.2E-9) in survival-associated CpGs in ER+ (Fig 4A). Furthermore, TR-ER_Q6 was enriched 1.24-fold (P = 0.04) in hazardous CpG regions and TR-ER_Q6_02 was enriched 1.18-fold (P = 0.05) in survival-associated CpG regions (protective and hazardous CpG regions combined) in ER- samples.

Next, we stratified patient samples into p53+ and p53- groups and calculated the enrichment of TF binding motifs with particular focus on p53 TF binding motifs (Fig 4B). We identified JA-TP53 to be enriched 1.27-fold in protective CpGs regions only in p53- samples (P = 0.02) and enriched 1.24-fold in survival-associated CpG regions (P = 0.005) (Fig 4B). Likewise, the TR-P53_01 motif was enriched 1.48-fold in protective CpG regions (P = 3.8E-5) and 1.38-fold in survival-associated CpG regions (P = 5.13E-6) in p53- samples only (Fig 4B). In contrast to the other p53 motifs, TR-P53_02 was enriched 1.29-fold in p53+ samples (P = 0.006) and 1.24-fold in p53- samples (P = 0.05), both in protective CpG regions (Fig 4B). Taken together, our enrichment results from ER- and p53-stratified breast cancer patients sample show that the majority of ERα and p53 TF binding motifs are enriched in CpG regions in ER- and p53- samples, respectively. Presumably, the binding sites of key transcriptional regulators can become unbound and accessible to other factors once the activity of the key TF is ablated. Alternative factors may then bind to these open sites leading to misregulation of the associated genes, and contribute to cancer progression and clinical outcomes of patients.

### TF binding motifs enriched in functional CpG regions associated with histological subtypes of breast cancer

Breast cancer, like most other cancer types, exhibits a high degree of heterogeneity making it refractory to treatment. One approach to abrogate the effects of sample-to-sample variation is to classify tumors into subtypes, each with distinct genetic, molecular, and physiological features. Therefore, we aimed to resolve whether breast cancer subtypes determined by immunohistochemistry also exhibit differences in TF binding motif enrichment near survival-associated CpGs.

First, we calculated TF binding motifs enriched/depleted in survival-associated CpGs in each histological subtype of breast cancer (S3, S4, S7–S12 Tables). In summary, there are a total of 252 (178 TFs), 135 (85 TFs), and 247 (168 TFs) TF binding motifs that are enriched in protective, hazardous, and survival-associated CpG regions, respectively in at least one subtype at significance level P<0.01. In the opposite direction, 323 (217 TFs), 49 (41), and 305 (208 TFs) motifs were depleted in protective, hazardous, and survival-associated CpG regions, respectively in at least one subtype at the same significance level (See S13 Table for more details). The large number of identified motifs suggests that a variety of TFs contribute to breast cancer development and each TFs activity may or may not be important drivers depending on the subtype.

**Fig 5:**
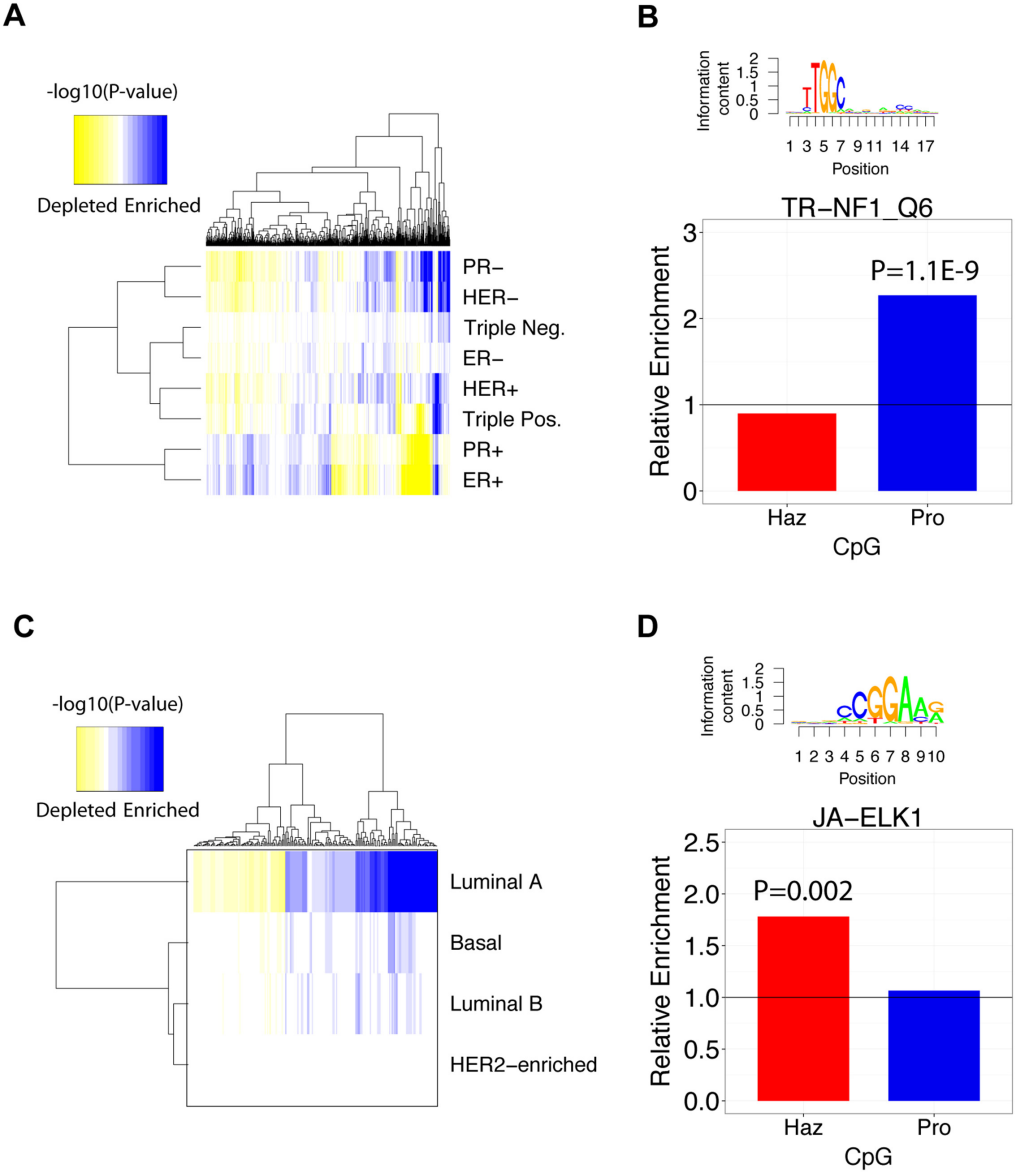
TF binding motifs enriched in histological and intrinsic subtypes of breast cancer. **A)** Hierarchical clustering of -log_10_(P-values) of TF motifs enriched in histological breast cancer subtypes. Only TF motifs enriched with P<0.05 were included in the clustering procedure. **B)** Enrichment values of TR-NF1_Q6 in triple-negative breast cancer. **C)** Hierarchical clustering of -log_10_(P-values) of TF binding motifs in intrinsic breast cancer subtypes. **D)** Enrichment values of JA-ELK1 in basal breast cancer. **A, C)** Enrichment of motifs is shown in blue and depletion is shown in yellow.

Second, we clustered the p-values (P<0.05) of significantly enriched or depleted TF binding motifs in survival-associated CpGs and observed that PR+ and ER+, which clustered together, exhibited enrichment patterns much different from that of the other subtypes (Fig 5A). More specifically, these subtypes are enriched in TF binding sites that are depleted in the other subtypes and vice versa. This suggests that the TFs associated with survival in PR+ and ER+ samples may not be significant protein factors in the other subtypes. In addition, it is clear that significantly enriched/depleted TF binding motifs vary from subtype to subtype implying that each subtype exhibits distinct TF-DNA methylation interactions. This shows that unique enrichment signatures can differentiate between breast cancer subtypes by revealing transcriptional regulators most likely to exhibit altered activity.

After showing global TF binding motif enrichment patterns of histological subtypes, we provide an example where the TR-NF1_Q6 motif is enriched 1.76-fold in protective CpG regions (P = 1.1E-9) in the triple-negative subtype (Fig 5B). Mutations in NF-1 have been implicated in the proliferation of triple-negative primary breast cancer tumors since it functions as an inhibitor of RAS and mTOR [52–54]. This suggests that DNA methylation within NF-1 binding sites is associated with longer survival times in patients with triple-negative breast cancer.

### Enrichment of TF binding motifs in functional CpG regions in intrinsic subtypes of breast cancer

To determine if different transcriptional regulators could also be identified in breast cancer subtypes based on molecular features, we classified our samples into 5 distinct intrinsic subtypes: luminal A, luminal B, HER2-enriched, and basal [55]. In some cases, intrinsic subtyping is more representative of the underlying molecular architecture in breast cancer and can be used to predict risk of cancer relapse after treatment with chemotherapy [56].

In our analysis, we first identified CpGs that were correlated with survival for each intrinsic subtype and determined which of 704 TF binding motifs were enriched in hazardous or protective CpG regions (S14–S16 Tables). In summary, a total of 9 (6 TFs), 209 (80 TFs), 113 (62 TFs) motifs were significantly enriched in protective, hazardous, and survival-associated CpGs, respectively (in at least one subtype P<0.01). Furthermore, 21 (16), 31 (27), and 40 (34) motifs were significantly depleted in protective, hazardous, and survival-associated CpGs, respectively (in at least one subtype P<0.01) (See S13 Table for more details).

Second, we clustered the enrichment p-values of significant TF binding motifs (P<0.05) in each intrinsic subtype and noticed that the luminal A subtype contained the largest number of significantly enriched/depleted TF binding motifs that yielded P<0.01 (Fig 5C). Conversely, HER2-enriched samples contained no significant TF binding site enriched or depleted in survival-associated CpG regions. This disparity is most likely due to differences in statistical power resulting from unequal subtype sample sizes and/or longer average patient survival times associated with different subtypes (Fig 5C). Despite this, it is clear that some enriched/depleted TF binding motifs are shared amongst luminal A, luminal B, and basal samples and some are not. Overall, this demonstrates global variation in TF binding site enrichment across intrinsic breast cancer subtypes.

To explore individual TF binding motifs that are enriched in an intrinsic subtype, we illustrate JA-ELK1 as an example. JA-ELK1 is enriched 1.78-fold in hazardous CpG regions in the basal subtype (P = 0.002) (Fig 5D). ELK1 has been shown to be involved in up-regulation of Mcl-1, a p53 inhibitor, and may contribute to survival of breast cancer cell lines [57]. Additionally, genome-wide studies in breast cancer cell lines have revealed that ELK1 is involved in the activation of c-Fos, a proto-oncogene that is implicated in tumorigenesis [58]. These studies verify that many TF binding motifs we find to be enriched in breast cancer subtypes are biologically meaningful in the context of breast cancer.

### TF binding motifs are enriched in different CpG clusters

**Fig 6:**
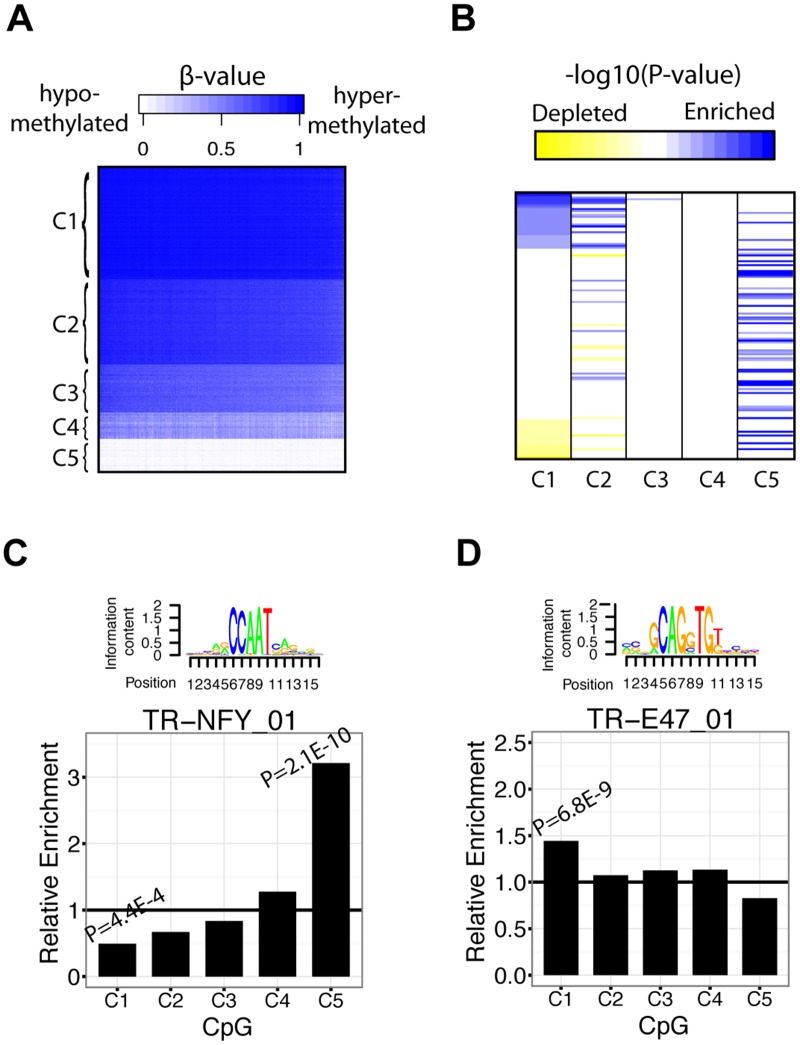
TF enriched in CpG subtypes of breast cancer. **A)** K-means clustering of CpG β-values into 5 distinct clusters. **B)** Ordering of significantly enriched/depleted (P<0.05) TF binding motifs relative to their -log_10_(P-value) ordering in cluster 1 (column 1). All -log_10_(P-values) greater than 10 were set to 10 and all values less than 3 were set to 0. Enrichment of motifs is shown in blue and depletion is shown as yellow. **C)** Enrichment values of TR-NFY_01 in each CpG subtype (C1–C5) **D)** Enrichment values of TR-E47_01 in each CpG subtype (C1–C5).

When analyzing TF-DNA methylation relationships in breast cancer subtypes, we build upon conventional methods of cancer stratification. However, in order to analyze TF motif enrichment within a classification scheme focused on DNA methylation, we adopted a bottom-up approach by first classifying all CpGs into subtypes based on their intensity levels. Since many cancers show genome-wide changes in DNA methylation, this approach may be able to identify TFs that are directly related to distinct intensity levels of DNA methylation. Therefore, we created a class of subtypes based on the clustering of CpG β-values and calculated TF binding motif enrichment in these subtypes. Fig 6A shows CpGs organized into 5 clusters based on β-values, with high intensity clusters on top and low intensity clusters on the bottom. From C1 to C5, the clusters are enriched in 68 (50 TFs), 45 (31 TFs), 6 (6 TFs), 6 (5 TFs), and 87 (59 TFs) TF binding motifs, respectively (P<0.05) (Fig 6A). Furthermore, we identified 119 (80 TFs), 38 (24 TFs), 3 (3 TFs), 1 (1 TFs), and 10 (8 TFs) TF binding motifs that were significantly depleted from C1 to C5, respectively (P<0.05) (S18 Table). Like histological and intrinsic subtypes of breast cancer, certain TF binding motifs exhibit different levels of enrichment across CpG subtypes. To globally illustrate the variation in TF motif enrichment between CpG subtypes, we sorted significant motifs in cluster 1 (C1) (P<0.01) from most enriched to most depleted (Fig 6B). We then ordered the TFs in the other 4 clusters relative to those belonging to cluster 1 (Fig 6B). From this, it is clear that related clusters share common patterns of enrichment (i.e. patterns in cluster 1 are more similar to that of cluster 2 than cluster 5) (Fig 6B). Interestingly, cluster C1, which contains highly methylated CpGs, is both enriched and depleted in TF binding motifs (Fig 6A and 6B). In contrast, cluster C5, which contains lowly methylated CpGs, is characterized mainly by TF binding motif enrichment events and few TF binding motif depletion events. This suggests that TF binding is generally associated with reduced methylation levels. Additionally, clusters C3 and C4 contain very few high-significance enriched/depleted TF binding motifs, suggesting that mid-intensity methylation are stochastic events and are not as informative for identifying important breast cancer-associated regulators.

To provide an example, we illustrate TR-NFY_01, which shows highest enrichment in C5 and lowest enrichment in C1 (Fig 6C). It can also be observed that its enrichment level increases from C1 to C5 (Fig 6C). This suggests that these CpG clusters have functional relevance in the context of NF-Y binding. NFY is known to be essential for proper cell cycle regulation and mutation of this protein can lead to inhibition of Cyclin A, RNR R2, DNA polymerase, CDC2, Cyclin B, and CDC25C [59]. Moreover, Agostino et al. showed that NF-Y facilitates gain-of-function p53 mutant binding to NF-Y promoters, resulting in cell cycle misregulation in breast cancer cell lines [60]. We also highlight TR_E47_01, which exhibited highest enrichment levels in C1 and lower enrichment levels in clusters least similar to C1, suggesting that E47 binding sites tend to be highly methylated in breast cancer (Fig 6D). E47 (also known was TCF3) is a repressor of E-cadherin and its activity has been implicated in epithelial-mesenchymal transition events in breast cancer [61].

### Network view of regulators associated with ER-stratified breast cancer

**Fig 7:**
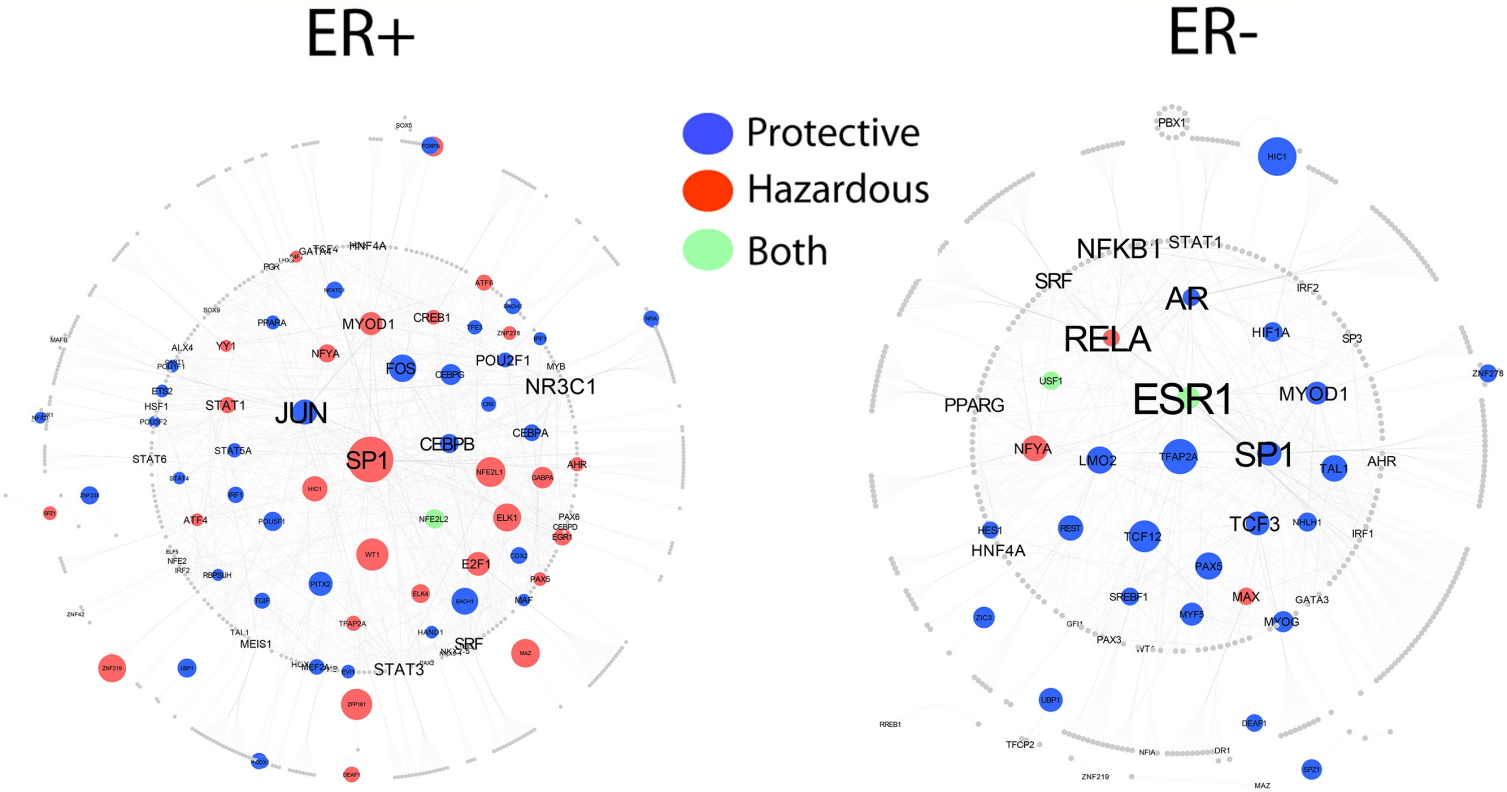
PPI network for TFs with binding motifs enriched in ER+ and ER- breast cancer. Only TFs with TF binding motifs enriched (depleted TF binding motifs excluded) with P<0.05 were included in the networks. Node circumference is scaled according to mean P-value of TF binding motifs of TFs. Larger nodes correspond to lower P-values. Text size is mapped to node degree where larger text font scales with higher node degree. Node color corresponds to the CpG category TF binding motifs are enriched in. Blue nodes indicate TFs with motifs enriched in protective CpG regions, red nodes indicate TFs with binding motifs enriched in hazardous CpG regions, and green nodes correspond to TFs with binding motifs enriched in both hazardous and protective CpG regions. Grey nodes are first-degree protein partners that interact with TFs whose motifs were identified as enriched in survival-associated CpG regions.

In order to demonstrate differences in the regulatory interactomes of breast cancer subtypes, we constructed two TF-TF interaction networks for ER+ and ER- samples (see Materials and Methods). Each network illustrates the first order partners of TFs whose motifs are significantly enriched (depletion is excluded) (P<0.01) in ER+ and ER- samples (Fig 7). Interestingly, in ER- breast cancer, ESR1 (ERα), RELA, SP1, and AR exhibit the highest degree in the network (Fig 7). Consistent with our prior results, it can be observed that ESR1 is significantly enriched in protective and hazardous CpGs in the ER- network only (Fig 7). In addition to ESR1, SP1 also exhibits high-degree in both ER+ and ER- networks; however, it is enriched in hazardous CpG regions in ER+ whereas, in ER-, it is enriched in protective CpG regions (Fig 7). This demonstrates that TF-DNA methylation relationships vary depending on disease context.

## Discussion

The effects of DNA methylation are widespread and vary according to genomic context and interactions with TFs. In this study, we proposed a novel method of inferring TF-DNA methylation relationships in breast cancer by utilizing both differential methylation and survival analysis to pinpoint informative CpGs. From these CpGs we were able to delineate TF involvement with methylation patterns and extend that to patient prognosis. Many mechanisms by which DNA methylation interacts with TFs have been proposed. It has been suggested that methylated CpGs can act as a direct physical hindrance to TF binding and thus interfere with its regulatory functions. Additionally, DNA methylation can recruit chromatin remodelers (or proteins that then recruit chromatin remodelers) to compact chromatin, assist in transcription elongation, or merely act as a passive marker of protein binding. This variety of potential mechanisms is key to understanding why the TF binding motifs of some experimentally verified oncofactors (oncogenic TFs) in our analysis were enriched in protective regions and vice versa for tumor suppressor motifs. For example, if methylated CpGs in the binding site of an oncofactor obstructs binding, then hypermethylation will ablate the oncofactor’s regulatory effect and promote survival. Alternatively, it is also possible for tumor suppressor TF binding motifs to be enriched in protective CpG regions if the role of the tumor suppressor is to recruit DNA methyltransferases to silence oncogenes. Additionally, the genomic location of CpGs may have an effect on its regulatory activity; methylated CpGs in promoters may inhibit gene expression but methylated CpGs in gene bodies may aid in transcriptional elongation [27]. Therefore, genomic context and prior knowledge of the TFs relationship with methylated CpGs must be established before reasonable conclusions can be made. As more experimental data is generated regarding these relationships, a TF-DNA methylation interactome network will be of greater use. Here, we have provided evidence that the application of motif enrichment, survival analysis, and differential methylation analysis can be integrated and used to define TF-DNA methylation interactomes in various subtypes of breast cancer. All in all, this study links TF-binding to DNA methylation to overall patient prognosis. By embracing the complexity of misregulation in various breast cancer subtypes, it may be possible to identify key players responsible for cancer subtypes and use that information to guide the development of treatment regimens in the clinic.

Furthermore, our preliminary analysis shows that correlating gene expression with survival yields very few significant genes after multiple hypotheses testing correction (S19 Table). This can be due to post-transcriptional modifications that affect the stability of mRNA transcripts, the fact that mRNA abundance is not always a good proxy for protein activity, and the short lifespans of cancer patients in our datasets. In light of this, the alternative use of DNA methylation signals can reveal significant CpGs even after testing corrections. This may be due to the fact that it is a binary chemical modification that is stable and can, in some cases, better reflect regulatory activity.

The most striking result of our analysis is that the majority of ERα and p53 TF binding motifs are significantly enriched in survival-associated CpG regions in ER- and p53- samples, respectively. From these results, we propose that ERα TF binding motifs that are not bound by their respective TF (as in the case of ERα in ER-samples) may become bound by alternative factors that may cause misregulation of downstream genes and impact patient survival. Indeed, this may also be the case for p53 in p53- samples. The reasoning for this model begins with the observation that the TF binding motifs of a master regulator are enriched in CpG regions whose methylation is correlated with survival only in samples missing that regulator. If a TF is missing, why would a significantly large proportion of its binding sites be enriched in these informative CpG regions? Therefore, we suspect that these motifs are open to binding by alternative factors whose binding events may simultaneously cause gene misregulation and detectable alterations in DNA methylation. Additionally, an alternative explanation may be that DNA methylation in p53 or ERα binding sites passively reflects the lack of p53 or ERα activity, respectively. This is valid in the case of p53- samples, where the loss of a key tumor suppressor would result in longer survival times compared to p53+ patients. However, in the case of ERα, its exact cancer regulatory roles are not as clear and thus difficult to interpret.

To explore breast cancer TF-DNA methylation relationships in depth, we adopted three different classification schemes by which we divide samples based on histological, molecular, and methylation features. Since each subtyping method utilizes information at different levels (i.e protein, gene expression, methylation) it is sensible to adopt all three strategies. By calculating TF binding motif enrichment in different subtypes, we can effectively determine the similarities and differences in their TF-DNA methylation signatures. We also observe differential enrichment patterns between protective and hazardous CpG sets among histological and intrinsic breast cancer subtypes, suggesting that TF-DNA methylation relationships vary across subtypes.

We also complemented our analyses by calculating whether TFs whose motifs were enriched in differentially methylated or survival-associated CpG regions were also differentially expressed in mRNA levels between ER+ and ER-, and between normal and tumor patient samples, respectively. We found that many of TFs with enriched motifs were also significantly differentially expressed, suggesting a greater biological role for these TFs (S1–S12 and S14–S17 Tables). Additionally we validated many of our identified TF binding motifs in an independent DNA methylation dataset published by Dedeurwaerder et al. [62]. This dataset was generated from the Illumina HumanMethylation27K (HM27K) array which profiled ~27,000 CpG sites from 248 primary breast tumors. Particularly we found that the TR-ER_Q6 and TR-ER_Q6_02 motifs were both significantly enriched ~1.3-fold (unadjusted P = 0.02 and P = 0.008, respectively) in hypomethylated (ER+<ER-) CpG regions between ER+ (n = 132) and ER- (n = 101) samples (S1 Table). Moreover, we found that GATA3 and FOXO1 TF binding motifs were enriched in hypomethylated regions (P<0.05), which is consistent with results from the main TCGA dataset. We then extended our validation analysis to include the enrichment calculation of TF binding motifs in survival-associated CpG regions identified across all breast tumor samples. In particular, we sought to confirm our enrichment results for the top 20 protective and top 20 hazardous TF binding motifs in the Dedeurwaerder dataset, and were able to validate 19 out of the 20 protective and 16 out of the 20 hazardous TF binding motifs (P<0.05, S2 Table). To note, we did not perform the CpG filtering procedure in the Dedeurwaerder dataset since the number of CpGs interrogated by the Illumina HM27K array was substantially less than the Illumina HM450K array used by TCGA, resulting in a sizeable decrease in statistical power. Together, these results indicate that our analysis remains robust across independent datasets even when different genomic platforms are used.

We concede that there are limitations to the informativeness and interpretation of our results. First, we used a 102 bp region to define a CpG region, which restricts our analysis to a local binned area. Even though most binding events only encompass ~100 bp, it may be possible that this sequence space may encompass the binding sites of TFs that are not associated with the CpG residue leading to false positives. On the other hand, the 102 bp region may be too small and not encompass the binding sites of TFs that do in fact interact with the CpG resulting in false negatives in our enrichment analysis. Overall, we experimented with varying sequence region sizes and determined that our results remain stable. Second, because we restricted our analysis to a local region, we do not take into account any potential long-range effects CpG sites may have on TF binding as a result of chromatin orientation. Third, limitations in platform technology must also be taken into account since only 450,000 out of a total of ~30 million CpGs are probed by the Illumina HumanMethylation 450K array. Fourth, we acknowledge that there may be differences in statistical power when conducting enrichment analysis in CpG sets (e.g. protective, hazardous) since the number of CpGs in each set may vary. Lastly, many of the analysis steps in our methodology including motif detection and setting significance criteria for survival-associated CpGs suffer from high false positive rates. However, we were able to overcome these obstacles by calculating relative enrichment of TF binding motifs. Therefore, even though we were able to identify significantly enriched TF binding motifs, our enrichment scores were ultimately biased towards the null (RE = 1). Overall, we maintain that our method has produced results that provide new insight into TF-DNA methylation relationships in breast cancer despite these limitations.

In this study, we have developed a novel method for identifying transcriptional regulators involved in breast cancer in the context of patient survival by using DNA methylation data derived from primary breast tumor tissue. By doing so, we have provided insight into the complexity of TF-DNA methylation interactomes that underlie breast cancer across a wide variety of subtypes. Our analysis has revealed several informative results and, in addition, raises a manifold of new questions regarding cancer misregulation. Namely, we have identified transcriptional regulators that affect patient prognosis and proposed a new model whereby breast carcinogenesis may be driven via binding of alternative factors to unbound TFBSs. Additionally, we considered the heterogeneity exhibited by breast cancer tumors by characterizing TF-DNA methylation relationships in histological, molecular, and DNA methylation subtypes. In this analysis, we focused on well-defined TF binding motifs, but it is also possible to combine this analysis with *de novo* motif identification to identify novel motifs that are enriched in differentially methylated or survival-associated CpG regions. Such integration could also allow for an exhaustive and systematic identification of non-TF regulators that may also interact with methylated CpGs (e.g. non-coding RNAs). Ultimately, our study has provided deep insight into the differential regulatory wiring of breast cancers that occur due to the divergent and combinatorial effects of diverse mutations.

## Materials and Methods

### DNA methylation data for primary breast cancer samples

Breast invasive carcinoma (BRCA) Level 3 DNA methylation datasets for the JHU-USC HumanMethylation450K platform, CpG annotation files, and clinical information were downloaded from the TCGA data portal [20]. CpG methylation signal intensities were represented as β-values in the datasets. In addition, Level 3 TCGA UNC AgilentG4502A_07 mRNA expression data was downloaded from the site data portal [20]. Subtype classification for all patient samples was derived from TCGA clinical information [20]. Breast cancer DNA methylation data and clinical information from Dedeurwaerder et al. was downloaded from the Gene Expression Omnibus (GEO) under the accession number GSE20713. PWMs for human TFs were obtained from the TRANSFAC [63] and JASPAR [64] databases. In some cases there were multiple PWMs for a single TF. The TF-TF physical interaction data were compiled from two resources: an experimental dataset from Ravsi et al [65] containing 5238 TF-TF physical protein interactions across 1400 human TFs, and the human protein reference database [66].

### Identification of differentially methylated CpGs

Patient samples that were accompanied with histological information regarding ER status were split into ER+ (405 samples) and ER- (122 samples) groups. Differentially methylated CpGs were then identified using two-tailed student t-test. To achieve stringency while maintaining power for enrichment analysis, a Benjamini-Hochberg adjusted P<0.05 was chosen as the cutoff for differentially methylated CpGs. CpGs with p-values below the cutoff with a t-statistic >0 and <0 were categorized into hypermethylated (ER+>ER-) and hypomethylated (ER+<ER-) sets, respectively. A Wilcoxon ranked sum test was also implemented to identify differentially methylated CpG sites.

### Identification of differentially expressed genes

RNA-seq data for 1154 breast cancers was downloaded from the TCGA data portal. Differentially expressed genes were identified using a Wilcoxon ranked sum test. A fold change >1 indicated gene up-regulation and a fold change <1 indicated down-regulation. Differentially expressed genes corresponding to TFs were included in all supplementary tables to complement TF enrichment information.

### Identification of CpGs associated with patient survival in all samples

The β-values for 376,667 CpGs across 562 samples and clinical data were used as input into a univariate Cox proportional hazards model [40]. Each CpG was considered individually and used as the covariate in the model. Significance of model coefficients was calculated using the Wald test. CpGs that yielded unadjusted P<0.02 and a hazard ratio of <1 or >1 were labeled as protective and hazardous, respectively.

### Identification of CpGs associated with patient survival in breast cancer subtypes

Samples were categorized into histological and intrinsic subtypes based on the clinical information downloaded from TCGA. The β-values for 376,667 CpGs across subtype-only samples were used as input into the Cox proportional hazards model where each CpG was considered individually. This allowed for the identification of survival-associated CpGs significant in the particular subtype.

### Identification of TF binding motifs

The 102 bp sequence region centered at each significant CpG was used as input into the FIMO software package [67] from the MEME suite to identify the existence of a motif in the region. Default parameters were used and a threshold cutoff of P<1E-4 was used to determine the presence of a motif. This yielded a matrix containing Boolean values indicating if a particular TFBS motif was present in a CpG region.

### *De novo* identification of TF binding motifs in hypermethylated and hypomethylated CpG regions

The top 10,000 most significant differentially methylated CpG regions (hyper- or hypo-) were chosen as input into the Discriminative Regular Expression Motif Elicitation (DREME) algorithm (MEME suite) using default parameters, with the exception of the maximum motif size, which was set to 20 [68]. Identified motifs were then queried against the JASPAR vertebrate database using Tomtom (MEME suite) to identify their cognate TFs [69].

### Filtering of overlapping CpG regions

Because overlapping CpG regions can lead to over-counting of TF binding motifs, we filtered out all overlapping CpG regions in each chromosome for forward and reverse DNA strands. The filtering procedure proceeds as such: **(*i*)** CpGs located on different chromosomes were considered non-overlapping. **(*ii*)** CpGs that were located on different DNA strands were considered non-overlapping. **(*iii*)** Sort all CpGs based on their genomic coordinates and identify clusters of CpGs with overlapping 102bp regions. **(*iv*)** For each cluster, identify the CpG with the lowest p-value (differentially methylated or survival-associated depending on analysis) and set this as the reference CpG. **(*v*)** Identify all within-cluster CpGs whose regions do not overlap with that of the initial reference CpG and filter out the rest. **(*vi*)** Of the non-overlapping CpGs, select the one with lowest P-value and set as the new reference CpG **(*vii*)** Iterate until all CpGs have either been selected or filtered out. (All “reference” CpGs are then included in the subsequent motif enrichment analysis.)

### Enrichment analysis

To compute enrichment of TFs in functional CpGs (survival-associated, differentially methylated, or clustered CpGs), we applied a two-sided Fisher’s exact test for each TF binding motif to determine if it was overrepresented or underrepresented in a CpG set. The Fisher’s exact test involves calculating the hypergeometric probabilities of all possible matrices of a 2X2 contingency table while keeping the margin counts fixed. The probabilities of all possible fixed-margin contingency tables more extreme than the current table were summed to determine the probability of over-representation/under-representation of a motif to occur by random chance. The R function “fisher.test” was used to implement this computationally. Enrichment was calculated in protective, hazardous, and survival-associated (protective & hazardous) CpG sets for histological, intrinsic, and CpG (CpG clusters) breast cancer subtypes. Additionally, motif enrichment was implemented in differentially methylated CpGs between ER+ and ER- breast cancers. The Benjamini-Hochberg multiple hypothesis testing correction procedure [70] was used to adjust the P-values outputted by multiple Fisher’s exact tests. All P-values presented in the Results section had been adjusted for multiple testing.

When comparing the distributions of TF binding motif enrichment values between hyper- and hypomethylated CpGs (Fig 2A), we first controlled for the potential effects that sample size may have on the power of enrichment analysis. This issue may arise due to the unequal number of CpGs belonging to hyper- and hypomethylated CpG sets. Therefore, we took the top *n* most significant CpGs from each set, where *n* is the smallest number of CpGs between the two sets, and carried out enrichment analysis.

### Construction of ER+ and ER- regulatory networks

TF binding motifs with P<0.01 in ER+ and ER- samples, and their first-order interacting partners were extracted from the TF-TF physical interaction network and used as input into Cytoscape to construct a regulatory network. TF network analysis was implemented using the “NetworkAnalyzer” function included in the software. The size of network nodes were mapped to the enrichment P-values of TFs represented in the network (lower P-values correspond to larger nodes). The font sizes of TF names were mapped to node degree in the network (larger font sizes correspond to higher degree). These mappings were implemented using Cytoscape’s VizMapper tools. If multiple motifs belonging to the same transcription factor fell below the significance threshold, their p-values were averaged.

### Clustering analysis of CpG sites

K-means clustering was applied to cluster CpGs based on their β-values. To determine the number of clusters, k-means was applied using 1–10 clusters and the total within-cluster sum of squares (WCSS) was calculated and graphed. Classification into 5 clusters yielded the last point where there is noticeable decrease in total WCSS.

All statistical and computational analyses were implemented in the R statistical programming environment.

## Supporting Information

S1 Table Enriched/Depleted TF binding sites in differentially methylated CpG regions between ER+ and ER- samples. (XLS)

S2 Table Enrichment/Depletion of TF binding motifs in survival-associated CpG regions in all breast cancers. (XLS)

S3 Table Enrichment/Depletion of TF binding motifs in survival-associated CpG regions in ER+ breast cancers. (XLS)

S4 Table Enrichment/Depletion of TF binding motifs in survival-associated CpG regions in ER- breast cancers. (XLS)

S5 Table Enrichment/Depletion of TF binding motifs in survival-associated CpG regions in p53+ breast cancers. (XLS)

S6 Table Enrichment/Depletion of TF binding motifs in survival-associated CpG regions in p53- breast cancers. (XLS)

S7 Table Enrichment/Depletion of TF binding motifs in survival-associated CpG regions in PR+ breast cancers. (XLS)

S8 Table Enrichment/Depletion of TF binding motifs in survival-associated CpG regions in PR- breast cancers. (XLS)

S9 Table Enrichment/Depletion of TF binding motifs in survival-associated CpG regions in HER2+ breast cancers. (XLS)

S10 Table Enrichment/Depletion of TF binding motifs in survival-associated CpG regions in HER2- breast cancers. (XLS)

S11 Table Enrichment/Depletion of TF binding motifs in survival-associated CpG regions in triple-positive breast cancers. (XLS)

S12 Table Enrichment/Depletion of TF binding motifs in survival-associated CpG regions in triple-negative breast cancers. (XLS)

S13 Table Number of enriched TF binding motifs in histological and intrinsic subtypes. (PDF)

S14 Table Enrichment/Depletion of TF binding motifs in survival-associated CpG regions in luminal A breast cancers. (XLS)

S15 Table Enrichment/Depletion of TF binding motifs in survival-associated CpG regions in luminal B breast cancers. (XLS)

S16 Table Enrichment/Depletion of TF binding motifs in survival-associated CpG regions in basal breast cancers. (XLS)

S17 Table Enrichment/Depletion of TF binding motifs in survival-associated CpG regions in HER2-enriched breast cancers. (XLS)

S18 Table Enrichment/Depletion of TF binding motifs in survival-associated CpG regions in CpG clusters. (XLS)

S19 Table mRNA transcripts that are correlated with breast cancer patient survival. (XLS)
