# Supplementary material for: Regulators Associated with Clinical Outcomes Revealed by DNA Methylation Data in Breast Cancer
Source: PLoS Comput Biol. 2015 May 21;11(5):e1004269. doi: 10.1371/journal.pcbi.1004269 (PMC4440643; doi:10.1371/journal.pcbi.1004269)
Supplement: S13 Table — (PDF) [file pcbi.1004269.s013.pdf]

Breast Cancer Subtype	Enriched (P≤0.01)			Depleted (P≤0.01)		
**Histological Subtypes**	**Protective**	**Hazardous**	**Pro+Haz**	**Protective**	**Hazardous**	**Pro+Haz**
Triple-positive	46 (30)	43 (27)	40 (26)	143 (98)	0 (0)	72 (54)
Triple-negative	41 (26)	0 (0)	14 (11)	12 (11)	0 (0)	1 (1)
PR+	69 (53)	32 (25)	37 (30)	135 (92)	0 (0)	106 (78)
PR-	116 (76)	30 (19)	128 (83)	129 (95)	0 (0)	135 (98)
HER+	36 (26)	106 (62)	67 (47)	54 (41)	15 (14)	61 (46)
HER-	91 (62)	32 (22)	89 (59)	117 (85)	2 (2)	76 (59)
ER+	102 (77)	99 (62)	80 (60)	161 (113)	7 (6)	150 (103)
ER-	69 (49)	14 (11)	10 (8)	21 (19)	28 (22)	14 (13)
**Intrinsic Subtypes**						
Basal	0 (0)	26 (16)	6 (4)	1 (1)	2 (2)	0 (0)
HER2-enriched	0 (0)	0 (0)	0 (0)	0 (0)	0 (0)	0 (0)
Luminal A	9 (6)	119 (72)	102 (61)	13 (11)	29 (25)	40 (34)
Luminal B	0 (0)	64 (42)	5 (4)	7 (5)	0 (0)	0 (0)
